# Association between Subcutaneous White Adipose Tissue and Serum 25-Hydroxyvitamin D in Overweight and Obese Adults

**DOI:** 10.3390/nu5093352

**Published:** 2013-08-26

**Authors:** Brian D. Piccolo, Gregory Dolnikowski, Elias Seyoum, Anthony P. Thomas, Erik R. Gertz, Elaine C. Souza, Leslie R. Woodhouse, John W. Newman, Nancy L. Keim, Sean H. Adams, Marta D. Van Loan

**Affiliations:** 1 Department of Nutrition & Graduate Group in Nutritional Biology, University of California, One Shields Avenue, Davis, CA 95616, USA; E-Mails: apthomas@ucdavis.edu (A.P.T.); elaine.souza@ucdmc.ucdavis.edu (E.C.S.); john.newman@ars.usda.gov (J.W.N.); nancy.keim@ars.usda.gov (N.L.K.); sean.h.adams@ars.usda.gov (S.H.A.); marta.vanloan@ars.usda.gov (M.D.V.L.); 2 Jean Mayer USDA-ARS, Human Nutrition Research Center on Aging at Tufts University, 711 Washington Street, Boston, MA 02111, USA; E-Mails: gregory.dolnikowski@tufts.edu (G.D.); elias_ephrem.seyoum@tufts.edu (E.S.); 3 Obesity & Metabolism Research Unit, USDA-ARS, Western Human Nutrition Research Center West Health Science Drive, Davis, CA 95616, USA; E-Mails: erik.gertz@ars.usda.gov (E.R.G.); leslie.woodhouse@ars.usda.gov (L.R.W.)

**Keywords:** vitamin D, 25(OH)D, obesity, adipose tissue, weight loss

## Abstract

Cholecalciferol is known to be deposited in human adipose tissue, but it is not known whether 25-hydroxyvitamin D (25(OH)D) is found in detectable concentrations. Therefore, our objective was to determine whether 25(OH)D is detectable in subcutaneous white adipose tissue (SWAT) in overweight and obese persons enrolled in a twelve week energy restricted diet. Baseline and post-intervention gluteal SWAT biopsies were collected from 20 subjects participating in a larger clinical weight loss intervention. LC-MS/MS was utilized to determine SWAT 25(OH)D concentrations. Serum 25(OH)D and 1,25(OH)_2_D were measured by RIA. Body composition was assessed by dual energy x-ray absorptiometry. SWAT 25(OH)D concentrations were 5.8 ± 2.6 nmol/kg tissue and 6.2 ± 2.7 nmol/kg tissue pre- and post-intervention SWAT, respectively. There was a significant positive association between SWAT 25(OH)D concentration and serum 25(OH)D concentration (*r* = 0.52, *P* < 0.01). Both SWAT and serum 25(OH)D concentrations did not significantly change after a twelve-week period of energy restriction with approximately 5 kg of fat loss. In conclusion, we have demonstrated our LC-MS/MS method can detect 25(OH)D_3_ in human subcutaneous fat tissue from overweight and obese individuals and is consistent with previously reported concentrations in swine. Additionally, our findings of no significant changes in SWAT 25(OH)D_3_ or serum 25(OH)D after a 6% loss of total body weight and 13% reduction in total fat provides the first human evidence that adipose 25(OH)D does not likely contribute to serum 25(OH)D with moderate weight loss; whether this is also the case with larger amounts of weight loss is unknown. Weight loss alone is not sufficient to increase serum 25(OH)D and increases in dietary or dermal biosynthesis of vitamin D appear to be the most critical contributors to in vitamin D status.

## 1. Introduction

The analysis of vitamin D metabolites in adipose tissue is rare and likely due to difficulties in extracting and separating the metabolites from tissue matrices [1]. Nonetheless, there is a disparity in the literature between analyses of the parent compound, cholecalciferol, and the other vitamin D metabolites in adipose tissue. This would be expected since it has been well-established that cholecalciferol is the dominant metabolite distributed in adipose tissue [2]. In fact, there are only two reports of measurable concentrations of 25(OH)D in adipose tissue from human cadavers [2] and in swine [3]; suggesting a small yet quantifiable site of 25(OH)D deposition in adipose tissue.

In a previous article, Heaney *et al*. [4] estimated adipose 25(OH)D content in a 70 kg woman with 24.5 kg fat mass and suggested a total of 114.5 nmol 25(OH)D was distributed within this tissue compartment. However, the data used for this estimation were from swine adipose tissue [3]. To our knowledge, there have been no reports of adipose 25(OH)D analyses in healthy human subjects. Although the Heaney estimation was derived from a non-human source, the authors make the argument that the total content of 25(OH)D is widely distributed within other tissue compartments. It is widely known that the highest tissue concentration of 25(OH)D is found in serum, however, adipose tissue content of 25(OH)D could exceed serum content due to its large overall mass. Therefore, the content of adipose 25(OH)D may need to be considered when determining total body vitamin D status.

Thus, we analyzed samples of subcutaneous white adipose tissue (SWAT) obtained from overweight and obese individuals participating in controlled feeding weight loss intervention to determine whether 25(OH)D can be measured in human adipose tissue. Our objectives were to determine ranges of 25(OH)D_3_ in human SWAT, to test if SWAT concentrations of 25(OH)D_3_ are altered as a result of weight loss, and to assess whether or not SWAT 25(OH)D correlates with blood levels of this metabolite in humans.

## 2. Materials and Methods

### 2.1. Study Design

The current study was exploratory and part of a larger human clinical trial investigating the inclusion of 3–4 servings of dairy foods in an energy restricted diet; details of the parent project have been previously published [5]. The clinical intervention trial was registered at clinicaltrials.gov (NCT 00858312). Healthy women, aged 20–45 years, and healthy men, aged 20–50 years (lower age for women was used to avoid hormonal changes associated with the transition to menopause that might affect endocrine parameters and related study outcomes), were enrolled in a 15-week controlled feeding study in which all foods were provided or prepared by the Metabolic Food Laboratory (MFL) at the USDA, ARS, Western Human Nutrition Research Center (WHNRC). The 15-week study was divided into a 3-week “Run-In” period during which subjects were weighed daily and caloric intake adjusted to maintain body weight; thereby establishing energy requirements. The “Run-In” period was followed by a 12-week “Weight Loss Intervention” during which energy intake was reduced by 500 kcal/day from the “Run-In” period. Therefore, pre-intervention measurements at the end of the “Run-In” period were considered baseline measures. Post-intervention measurements were made at the end of the 12-week “Weight Loss Intervention” period. Specific details regarding the dietary portion of the parent study can be found in the previously published manuscript [5]. The study and all procedures were approved by the Committee for Human Subjects Protection, Institutional Review Board of the University of California, Davis. Written informed consent was obtained from all research volunteers.

### 2.2. Subject Screening and Selection

Overweight and obese adult females and males were recruited from the faculty, staff, and student population at University of California, Davis as well as the greater Davis and Sacramento, CA communities. Body mass index was between 28 and 37 kg/m^2^. All participants were habitually low dairy consumers (defined as ≤1 serving/day), and weight stable (no more than 3 kg weight loss during three months preceding the intervention). Exclusion was based on not meeting the age, not meeting the dairy and calcium criteria (≤1 serving of dairy/day and a total calcium intake ≤ 600 mg/day from all sources), diagnosis of type 2 diabetes, fasting glucose ≥ 110 mg/dL, adverse response to study foods (lactose intolerance, dairy intolerance, dairy allergy determined by self-report), history or presence of significant metabolic disease (*i.e.*, endocrine, hepatic, or renal), use of blood pressure or lipid-altering medications, resting blood pressure ≥ 160/100 mg/Hg, triglyceride value ≥ 400 mg/dL or LDL ≥ 160 mg/dL, history of eating disorder, presence of active gastrointestinal disorders, pregnancy or lactation, use of obesity pharmacotherapeutic agents within the last 12 weeks, use of over-the-counter anti-obesity agents (e.g., those containing phenylpropanolamine, ephedrine and/or caffeine) within the last 12 weeks, use of calcium supplements in the past 12 weeks, recent (past four weeks) initiation of an exercise program, recent (past 12 weeks) initiation of hormonal birth control or change in hormonal birth control regimen, use of tobacco products, and exercise more than 30 min/day.

Body composition was assessed during the “Run-In” period and subjects were pair-matched based on percent body fat (%BF) and randomly assigned to one of two treatment groups: low dairy (LD, ≤1 serving/day) or adequate dairy (AD, 3–4 servings/day). Enrollment was continuous with a new cohort starting approximately every 8 weeks. Consequently, volunteers were enrolled year-round. The Consort Diagram providing information on the number of eligible participants *vs*. enrolled participants has been previously published [5]. Forty percent of the participants were non-white and 25% of those were of Hispanic origin.

### 2.3. Dietary Intake of Vitamin D

Vitamin D content of the diet was determined by the Nutrition Data System for Research software (NDSR software version 2011, University of Minnesota, Minneapolis, MN) and expressed as daily IU intake. Daily dietary vitamin D intake varied based on diet group (AD, LD) and the menu day for the 7 day rotating diet. Average daily vitamin D intake is shown in Table 1.

**Table 1:** Subject characteristics at Baseline and Post-Intervention ^1^.

Variable	Baseline	Post-Intervention
N	20	20
Sex (F/M)	15/5	
Age (y)	34.3 (9.0)	
Height (cm)	170 (1.0)	
Weight (kg)	93.1 (10.9)	87.3 (10.3) *
BMI (kg/m^2^)	32.7 (2.5)	30.6 (2.5) *
Total Fat Mass (kg)	38.5 (8.0)	33.5 (8.7) *
Body Fat (%)	43.5 (6.0)	40.9 (6.7)
Lean Mass (kg)	49.3 (10.0)	48.3 (9.6)
Waist Circumference (cm) ^2^	94.2 (8.3)	89.2 (7.2) *
Intra-abdominal Adipose Tissue, cc	41.7 (20.0)	31.5 (15.9) *
Android Fat (kg)	3.8 (0.7)	3.3 (0.7) *
Gynoid Fat (kg)	7.3 (1.5)	6.6 (1.5) *
Dietary Vitamin D intake (IU/day)	190.2 (18.2)	216.5 (83.8)

^1^ Values expressed as mean (SD) (except n and Sex); * denotes significant difference (*P* ≤ 0.05) between baseline and post-intervention based on paired t-test; ^2^ *n* = 19 post-intervention.

### 2.4. Body Composition Measurements

Body weight was measured on an electronic scale (Scale-tronic model 6002; Wheaton, IL, USA) to the nearest 0.1 kg with subjects in light clothing, all jewelry removed, pockets emptied, and shoes removed. Height was measured using a wall mounted stadiometer (Ayrton Stadiometer model S100; Prior Lake MN) and recorded to the nearest 0.1 cm. Body mass index was calculated as kg/m^2^. Waist circumference was measured with a metal non stretchable tape measure (Model Gluick, Lafayette Instrument, Lafayette, IN, USA) to the nearest 0.1 cm in the standing position against bare skin with the abdomen relaxed and arms at the sides. The average of two readings was reported for weight, height and waist circumference. Fat mass were assessed using dual energy x-ray absorptiometry (DXA, GE Lunar, Prodigy Model) during the “Run-In” period and at the end of the 12-week intervention. Daily calibration procedures were carried out per manufacturer instructions. To reduce the variance in the measurement data, all DXA scans were analyzed by a single technician. Intra-abdominal adipose tissue was measured using computed tomography (CT) trans-abdominal slices (Siemens Somaton 16 CT Scanner). Details of CT-scans have been previously published [5].

### 2.5. Serum Vitamin D

Fasting blood was drawn using sterile phlebotomony techniques at the end of the “Run-In” period and at the end of the 12 week intervention. Serum and plasma were stored at −80 °C until analyzed. Serum vitamin D metabolites [25(OH)D and 1,25(OH)2D] were analyzed using standard radioimmunoassay (RIA) procedures (DiaSorin, StillWater, MN and Immunodiagnostics Systems [IDS], Fountain Hills, AZ, respectively). Participants’ baseline and post-intervention samples were run simultaneously in duplicate and processed in accordance with manufacturer’s instructions. Interassay CVs were 6.0% and 6.3% for 25(OH)D and 1,25(OH)_2_D, respectively. In regard to the DiaSorin RIA, both 25(OH)D_2_ and 25(OH)D_3_ have 100% cross-reactivity with the internal antibody. Therefore, reported serum 25(OH)D values herein represent cumulative 25(OH)D_2_ and 25(OH)D_3_ concentrations. The WHNRC participates in the Vitamin D External Quality Assessment Scheme (DEQAS) and calibration standards of 25(OH)D and 1,25(OH)_2_D from this program were analyzed in conjunction with participant samples.

### 2.6. Subcutaneous White Adipose Tissue (SWAT) Biopsies

SWAT biopsies were obtained at the UC Davis Medical Center Clinical and Translation Science Center at “Run-In” and at the end of the 12-week intervention following a 12-h overnight fast. Biopsy samples from “Run-In” and at the end of the 12-week intervention were collected from opposite sides of the buttocks. Each sample was immediately rinsed in ice cold phosphate buffered saline (PBS) to minimize blood contamination. Samples were additionally rinsed in fresh ice cold PBS and aliquots were frozen in liquid nitrogen. Frozen samples were than stored at −80 °C until analyzed.

SWAT biopsies from participants enrolled in cohorts beginning in November through January were chosen for analysis to minimize potential influences by endogenous vitamin D synthesis. Fourteen subjects from winter cohorts had complete SWAT biopsy sets (both baseline and post-intervention) available, therefore, six subjects were randomly chosen from spring cohorts (enrollment between February and April) to bring the total number of SWAT samples analyzed to twenty pairs. Biopsies used for this study were shipped overnight on dry ice to the Jean Mayer USDA Human Nutrition Research Center on Aging at Tufts University for analysis.

### 2.7. Preparation and Analysis of SWAT 25(OH)D_3_ by LC-MS/MS

Subsamples of SWAT tissue (0.1–0.5 g) were added to a glass mortar filled with 2 g of 40 μm Bondesil-C18. Tissue was blended with a glass pestle and spiked with 0.2 mL mixture of 25-OH deuterated vitamin D_3_-d_6_ standards (Medical Isotopes, Inc., Pelham, NH, USA; purity 99.6%), each at 111 ng/mL. The mixture was further homogenized with a spatula and transferred to a 15 mL reservoir tube. A 20 μm frit was placed on top of the reservoir. The reservoir was tightly compressed with a 10 cc syringe plunger and 8 mL of water was added. The contents were collected and reduced to dryness using a Vac-Elute set to “Collect” mode. Residues were solubilized in acetone (4 mL), with supernatants being isolated, centrifuged at 3000 *g* for 11 min and filtered through a 0.2 μm PTFE filter. The extract volume was reduced under N_2_ at 45 °C for 60 min and 200 μL of the remaining extract was transferred to an autosampler vial insert. A 50 μL aliquot was injected on a LC-MS/MS (Agilent 1100 HPLC-ABSciex 5500 QTRAP tandem mass spectrometer). The HPLC was equipped with a 4.6 × 250 mm, 5-μm ProntoSIL 200-5-C30 (MAC-MOD Analytical, Inc., Chadds Ford, PA, USA, manufactured by Bischoff Chromatography, Leonberg, Germany) C-30 column. The column was maintained at room temperature during the analyses. The mobile phase flow rate was 0.9 mL/min. Solvent A was methanol and solvent B was methylene chloride. The gradient was: 0–15 min, 100% solvent A, followed by 50% solvent A from 10.0 to 25.0 min and a 10 min re-equilibrated at 100% A. Post-column solvent flow was diverted to waste during the final 13 min. The mass spectrometer employed an atmospheric pressure chemical ionization source operated in positive ion mode. The ion source temperature was 400 °C. The triple quadrupole MS/MS was operated in multiple reaction monitoring (MRM) mode to detect the mass transitions of the parent molecules after in source loss of water (*i.e.*, [M − H_2_O + H]^+^) for 25-hydroxy vitamin D_3_ *m*/*z* 383.4 > 211.3 and 25-hydroxy vitamin D_3_-d_6_ *m*/*z* 389.4 > 211.3, respectively. The collision gas was nitrogen and the pressure was set to medium. The collision energy for all MRM transitions was 40 V and the dwell time for each MRM transition was 1000 ms. As described above, we utilized isotope dilution procedures to internalize standards within samples [6,7]. By adding a known quantities of isotopically labeled metabolites vitamin D_3_-d_6_ to each sample, the isotope ratio of the resulting solution can be deconvoluted using the known isotope ratio of the labeled compounds and the natural deuterium abundance in the sample (<0.1%), to calculated the D_3_ metabolite concentrations in the samples. The average recovery was 72%.

Based on the average variance of low abundance calibration standards (2.5 nmol/kg), method limit of detection and limit of quantification were estimated at 1.0 and 2.9 nmol/kg, respectively. All reported data were greater than 2x the limit of detection estimate.

### 2.8. Statistical Methods

All statistical analyses were performed using R version 2.15.1 (R Development Core Team 2012). Univariate analysis, including mean, range and standard deviation, was used for baseline and post-intervention physical and clinical characteristics. Associations among variables were determined using Pearson’s correlation coefficients. Paired *t*-tests were used to determine differences between baseline and post-intervention measurements. All *P*-values ≤ 0.05 were considered statistically significant. Graphical plots were constructed using the R package, ggplot2 [8].

To minimize any potential effects of sun exposure, biopsy samples were specifically chosen from winter and spring cohorts. Consequently, the treatment assignment became unbalanced with 7 samples assigned to the AD group and 13 samples to the LD group. Additionally, by chance, 2 samples within the AD group had undetectable 25(OH)D, which reduced the number of paired baseline and post-intervention for the AD group to 5. Thus, treatment effect was not analyzed due to unequal and small sample sizes in each treatment group.

## 3. Results

### 3.1. Physical Characteristics

Physical characteristics of the subjects at pre- and post-intervention are shown in Table 1. The majority of subjects were female and 90% of the subjects were categorized as obese at baseline. Except for percent body fat, all adiposity markers significantly decreased by the end of the intervention. Participants lost an average of 6.1% total body weight and 12.8% total fat mass over the study period. Dietary vitamin D intakes were below current recommendations of 600 IU/day; however, the study was conducted during the previous recommendations of 200 IU/day. There was no statistical difference between baseline and intervention vitamin D intake. The large post-intervention standard deviation seen in vitamin D intake compared to baseline intakes was due to the inclusion of dairy foods for half of the subjects as part of the parent project intervention.

### 3.2. Serum and SWAT Analyses

Serum and SWAT concentrations of 25(OH)D and 1,25(OH)_2_D are shown in Table 2. Based on the guidelines suggested by the 2011 DRI for vitamin D, only two subjects had adequate serum 25(OH)D concentrations (≥50 nmol/L) at baseline. Of the remaining participants with sub-adequate serum 25(OH)D, eight had deficient serum 25(OH)D concentrations (<30 nmol/L). By the end of the intervention, the number of participants with adequate serum 25(OH)D concentrations improved in three participants, while the number of participants with deficient concentrations decreased by six participants. However, significant changes in serum vitamin D metabolites were not detected for the overall group.

**Table 2:** Serum and Subcutaneous White Adipose Tissue concentrations of 25(OH)D and 1,25(OH)_2_D at Baseline and Post-Weight Loss Intervention in adults ^1^.

Variable	Baseline	Post-Intervention
Serum 25(OH)D, (nmol/L)	33.5 (12.0)	38.6 (10.3)
Serum 1,25(OH)_2_D (pmol/L)	108.4 (21.5)	105.8 (30.6)
SWAT 25(OH)D_3_ (nmol/kg tissue) ^2^	5.8 (2.6)	6.2 (2.7)

^1^ Values expressed as mean (SD); ^2^ *n* = 18 baseline, 2 SWAT samples below detection; *n* = 19 post-intervention, 1 SWAT sample below detection.

SWAT 25(OH)D_3_ concentrations were detected in 18 baseline samples and 19 post-intervention samples. Concentrations ranged from 2.3 to 12.8 nmol/kg SWAT (0.9–4.9 ng/g) at baseline and 2.8–13.1 nmol/kg SWAT (1.1–5.03 ng/g) post-intervention. There was no statistical difference between baseline and post-intervention SWAT 25(OH)D_3_ concentrations (Table 2).

**Figure 1:**
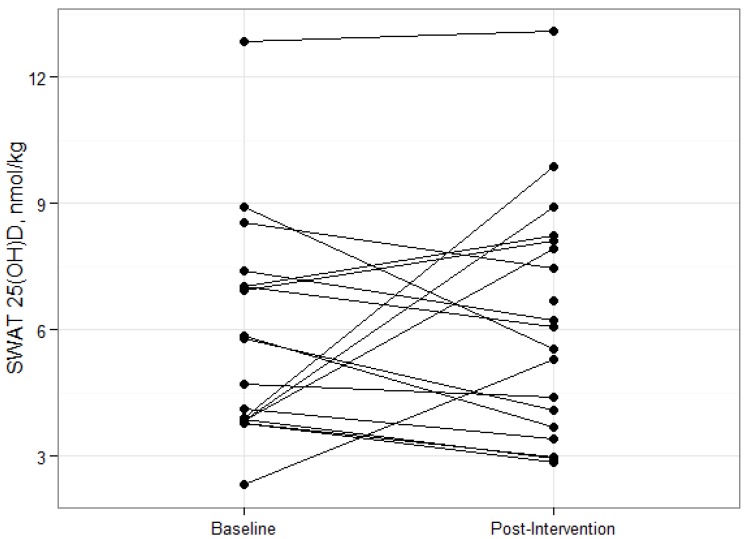
Individual changes in Subcutaneous White Adipose Tissue 25(OH)D_3_ concentrations from Baseline to Post-Intervention in adults who participated in a 15 week weight loss trial. *n* = 18 (Baseline) and 19 (Post-Intervention).

**Figure 2:**
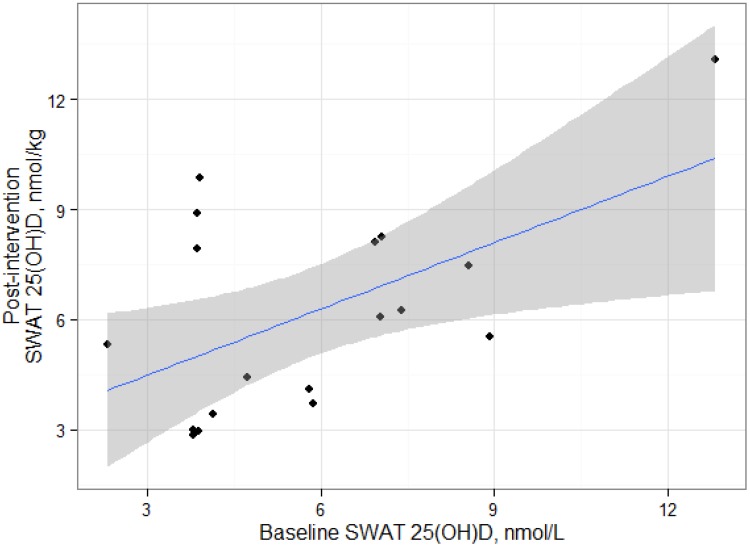
Association between Subcutaneous White Adipose Tissue 25(OH)D_3_ at Baseline and Post-Intervention in adults who participated in a 15 week weight loss trial. Shaded line = 95% Confidence Interval.

**Figure 3:**
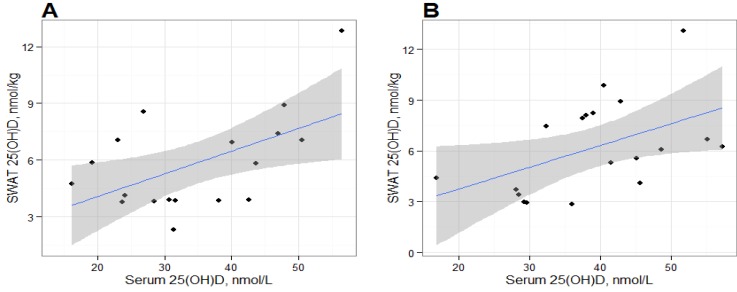
Association between Subcutaneous White Adipose Tissue and Serum 25(OH)D at (**a**) Baseline and (**b**) Post-Intervention following a 15 week weight loss regimen in adults. Shaded area = 95% Confidence Interval.

Individual changes in SWAT 25(OH)D_3_ concentrations varied (Figure 1) and ranged from −3.4 nmol/kg to 6.0 nmol/kg. Baseline and post-intervention SWAT 25(OH)D_3_ concentrations were significantly correlated (Figure 2; *r* = 0.55, *P* = 0.02) and SWAT and serum 25(OH)D concentrations were significantly correlated at baseline (*r* = 0.55, *P* = 0.02) and post-intervention (*r* = 0.48, *P* = 0.04) respectively (Figure 3). The absolute change of serum and SWAT concentrations were not correlated (*r* = −0.13, *P* = 0.60). Additionally, serum 1,25(OH)_2_D concentrations and SWAT 25(OH)D_3_ concentrations were not significantly correlated (*r* = −0.12, *P* = 0.49).

## 4. Discussion

Little is known about the content of 25(OH)D in human adipose tissue and whether weight loss or other conditions that impact adiposity influence fat tissue vitamin D metabolites. In swine, Jakobsen *et al*. found mean subcutaneous fat concentrations of 25(OH)D between 4.8 nmol/kg and 6.3 nmol/kg depending on the inclusion of vitamin D or 25(OH)D in their feed [3]. Our SWAT 25(OH)D_3_ concentrations of 5.8 nmol/kg at baseline and 6.2 nmol/kg post-intervention are in agreement with Jakobsen *et al*. and add to the few papers that have found measureable SWAT 25(OH)D concentrations [2]. However, it is acknowledged that other researchers have failed to detect 25(OH)D in adipose tissue [9]. This could be partly due to methodological differences. We quantified SWAT 25(OH)D_3_ using LC-MS/MS and acknowledge that at the time of our study there was no reference method to which our data could be compared. Considering the controversy regarding accurately assessing serum 25(OH)D concentrations [10,11,12,13], it would be presumptuous to relate our results to the general population. More analyses are needed to determine typical concentrations in wider populations of overweight and obese individuals and in different racial groups, and the current experiment provides a useful foundation for these efforts.

The majority of the subjects in our study had insufficient serum 25(OH)D concentrations according to the current IOM guidelines. This finding is consistent with other studies reporting an inverse relationship between measures of adiposity and serum 25(OH)D [14,15,16,17,18]. Deposition and sequestration of cholecalciferol in adipose tissue stores is an often cited mechanism behind this relationship, however there is very little experimental evidence to support this hypothesis. In this study, overweight and obese participants lost an average of 6% total body weight and 13% total fat. The fact that SWAT 25(OH)D_3_ concentrations remained unchanged after a significant decrease in adipose mass would suggest a release of 25(OH)D from adipose tissue. However, there were no changes in serum 25(OH)D, suggesting little contribution of released SWAT 25(OH)D to serum 25(OH)D concentrations. Drincic *et al*., [19] recently proposed that the inverse relationship between obesity and serum 25(OH)D is due to volumetric dilution of cholecalciferol in serum and adipose tissue. Perhaps, a similar mechanism exists between serum and adipose compartments of 25(OH)D. The extra hydroxyl group on 25(OH)D would make it slightly less hydrophilic and less dilutable in adipose tissue than cholecalciferol resulting in a smaller diffusional equilibrium. Therefore, the release of 25(OH)D due to the volumetric decrease in adipose tissue would result in an attenuated effect on serum 25(OH)D as seen in our study.

The subjects in our study were overweight and obese, but did not have metabolic disorders associated with excess adiposity such as type 2 diabetes, metabolic syndrome and other metabolic diseases (*i.e.*, renal disease). Therefore, we would not expect any perturbations in serum 1,25(OH)_2_D concentrations. Regulation of serum 1,25(OH)_2_D is tightly controlled and is generally not a good marker of vitamin D status [20]. Most of the serum 1,25(OH)_2_D is thought to be produced by renal 1-alpha-hydroxylase enzymes, but many non-renal tissues also contain 1-alpha-hydroxylase that convert 25(OH)D to 1,25(OH)_2_D for local gene regulation [21]. The supply of 25(OH)D is generally thought to be provided by the serum pool, but a small reservoir of 25(OH)D in the lipid droplets of adipocytes could supply the cell its functional need. *In vitro* evidence suggests that 1,25(OH)_2_D may regulate adipogenesis and lipid metabolism [22], however results from these studies have not been confirmed in human studies. It is an intriguing possibility to link 25(OH)D stored in lipid droplets to adipocyte gene regulation, but much more work still is needed.

We found positive and statistically significant relationships between serum 25(OH)D and SWAT 25(OH)D concentrations at baseline and post-intervention, suggesting that the deposition of 25(OH)D in SWAT could be dependent on serum 25(OH)D concentrations. Serum and adipose cholecalciferol concentrations have been previously reported to be positively correlated [23], but to our knowledge we are the first to report the relationship between serum 25(OH)D_3_ and SWAT 25(OH)D concentrations. The correlation between serum and SWAT 25(OH)D was similar at baseline and at post-intervention, suggesting an equilibrium between the two compartments. However, our results are based on alternative analytical methods for adipose tissue and serum 25(OH)D measurements which likely would affect the statistical interpretation. LC-MS/MS methods and the DiaSorin RIA generally produce consistent results in serum [24,25], but the DiaSorin RIA cannot distinguish between serum 25(OH)D_2_ and 25(OH)D_3_, while the LC-MS/MS adipose method was specific for only 25(OH)D_3_. Thus, we run the risk of under-reporting our SWAT 25(OH)D in comparison to serum concentrations. Serum concentrations of 25(OH)D_2_ are substantially lower than 25(OH)D_3_ [26,27,28] unless supplementing with vitamin D_2_ or consuming plant sources of vitamin D_2_. Based on the low 25(OH)D concentrations seen in our subjects, we would expect very little vitamin D_2_ consumption from supplements and, subsequently, very little 25(OH)D_2_ in adipose depots. Still, future studies should utilize analytical methods that can distinguish between both isoforms to accurately identify the relationship between the serum and adipose compartments.

It is important to recognize that the statistical significance of these correlations should be interpreted carefully due to the limited number of samples in this exploratory study. The statistical significance of each correlation appear to be leveraged by the participant with the greatest SWAT 25(OH)D_3_ measurement and removal of this datum results in non-significant correlations. It is unclear whether this participant is truly an outlier or would have been in the range of observed data from a larger sample. It should be noted that that our sample size is consistent with other studies that have measured cholecalciferol in humans [23,29,30], however, conclusions based on our results should be considered hypothesis generating data.

The changes in SWAT concentrations of vitamin D metabolites during energy restriction have not been reported. Our subjects lost an average of 5 kg of total fat mass after twelve weeks of reducing their energy requirements by 500 kcal, but had no significant change in SWAT, or serum, 25(OH)D concentrations. In contrast, serum cholecalciferol concentrations were shown to increase in fasted rats after the termination of a 14-day large daily vitamin D supplementation, [31], suggesting mobilization from adipose tissue stores. A recent study reported that women who lost more than 15% total body weight significantly increased serum 25(OH)D concentrations compared to women who were weight stable or lost ≤15% total body weight [32]. It is not definitively known in that particular study whether the increase in serum 25(OH)D concentration was the result of liberated cholecalciferol or 25(OH)D from adipose stores because no adipose biopsies were collected. Our subjects lost an average of only 6% total body weight after the intervention and we also failed to see any significant changes in serum 25(OH)D. The data from these studies suggest that a substantial increase in adipocyte lipolysis is required to free stored cholecalciferol or perhaps, 25(OH)D, to significantly increase serum 25(OH)D concentrations.

As discussed earlier, Heaney *et al*. hypothesized that a total of 114.5 nmol 25(OH)D was distributed in 24.5 kg of total fat in a hypothetical 70 kg woman [4]. This is assuming that the concentration of 25(OH)D is equally distributed throughout the adipose mass. Using the same approach in our subjects, we estimate mean total adipose 25(OH)D content at baseline and post-intervention to be 212.2 ± 93.7 and 199.6 ± 95.7 nmol, respectively. Additionally, estimated total fat 25(OH)D content ranged from 92.0 to 451.7 nmol, suggesting significant individual variability. Our samples were obtained from opposite gluteal sites and were relatively uniform between both time points. However, we cannot assume that the adipose 25(OH)D is distributed evenly in other adipose sites. Cholecalciferol deposition in adipose tissue has been shown to be quite variable among anatomical adipose sites [9,30] and there is not enough evidence to suggest either a uniform or variable distribution of fat 25(OH)D at this time.

The strengths of our study were the longitudinal collection of SWAT, and thus measurement of within subject changes. To our knowledge, the current study is the first to report 25(OH)D concentrations in SWAT pre- and post-weight loss in humans. Additionally, we analyzed samples across a broad range of body weight, adiposity, and serum 25(OH)D concentrations. By leveraging samples from a larger controlled feeding trial, it enabled us to accurately control and assess vitamin D intake, caloric restriction, and other parameters.

Due to the exploratory, proof-of-principle nature of this investigation and the cost associated with the analyses, we restricted our first study to a limited number of samples. Additionally, our subjects were derived from a larger study that did not control for factors that could introduce inter-subject variability in our analysis. The parent project introduced dairy products that are fortified with vitamin D, therefore subjects had variable intakes of vitamin D which could contribute to the variability in post-intervention measurements. We selected samples from winter and spring cohorts to minimize the possible effect of sun exposure, but inter-subject variability in serum and SWAT 25(OH)D_3_ measurements are likely attributed to seasonal differences. Also, serum analysis of 25(OH)D had previously been conducted for the parent study. Therefore, differences in methodological analyses could impact the accuracy of our measurements. Our subjects were considered overweight and obese yet generally healthy, so our findings may not apply to other overweight and obese individuals diagnosed with metabolic syndrome or other metabolic disturbances. Lastly, it is acknowledged that despite quite thorough biopsy washing steps, our samples may have minor contamination with blood, which could theoretically add variability to the outcome measurements and influence serum *vs*. SWAT correlations. Regardless, the current work provides a solid reference framework for understanding physiological concentrations of adipose 25(OH)D concentrations in humans.

## 5. Conclusions and Implications

In summary, we have demonstrated a LC-MS/MS method that can detect 25(OH)D in human adipose tissue collected from overweight and obese individuals enrolled in a clinical weight loss intervention. We found mean SWAT 25(OH)D concentrations of 5.8 ± 2.6 nmol/kg tissue and 6.2 ± 2.7 nmol/kg tissue pre- and post-intervention, respectively. Obese persons are thought to be at risk for vitamin D deficiency, which could contribute to the development or exacerbate other comorbidities associated with excessive adipose tissue. Although there are very little human data, the accumulation and release of vitamin D metabolites in excess adipose tissue is the mechanism often cited behind this relationship. Aside from the small 25(OH)D concentrations detected in SWAT, our results suggest that 25(OH)D is released from adipose tissue after a loss of 6% in body weight and a 13% reduction in total fat loss, but this release contributes little to serum 25(OH)D concentrations. Perhaps a greater loss of body weight or fat is needed to observe a significant contribution to serum 25(OH)D concentrations (*i.e.*, ≥15% loss). Therefore, dietary intake and dermal biosynthesis should be paramount when devising strategies to improve serum 25(OH)D in obese individuals.
